# The integration of immune checkpoint inhibitors with VEGF targeted agents in advanced gastric and gastroesophageal adenocarcinoma: a review on the rationale and results of early phase trials

**DOI:** 10.1186/s13045-021-01034-0

**Published:** 2021-01-12

**Authors:** Anwaar Saeed, Robin Park, Weijing Sun

**Affiliations:** 1 Division of Medical Oncology, Department of Medicine, University of Kansas Medical Center, 2330 Shawnee Mission Pkwy, Suite 210, Westwood, Kansas City, KS 66205 USA; 2 MetroWest Medical Center/Tufts University School of Medicine, Framingham, MA USA

**Keywords:** Combination therapy, Angiogenesis, Immune checkpoint inhibitor, Gastric cancer, Esophageal cancer, Gastroesophageal cancer, Programmed death-1, Programmed death ligand-1, Cytotoxic lymphocyte antigen-4, Vascular endothelial growth factor

## Abstract

Several targeted therapies have shown efficacy in patients with advanced gastric cancer (GC) and gastroesophageal junction adenocarcinoma (GEJC), including anti-angiogenic agents and immune checkpoint inhibitors. Ramucirumab, an anti-VEGFR2 antibody, has shown efficacy in GC, but the benefits are limited, in part due to MET-mediated resistance. Other VEGF targeted agents like VEGF tyrosine kinase inhibitors (TKIs) with broad multi-kinase inhibitory spectrum like regorafenib and cabozantinib have also shown modest single agent activity in early phase trials. For immune checkpoint inhibitors, pembrolizumab (anti-PD-1) monotherapy confers survival advantage as 3rd line therapy for the PD-L1 expressing GC and GEJC population and has been approved for use in this setting. Extensive tumor microenvironment immune modulatory effects from antiangiogenic agents have been demonstrated from preclinical data which support the clinical study rationale of dual blockade of VEGF and immune checkpoint. In addition, FDA has approved combinations of anti-VEGF/VEGFR with anti-PD-1/PD-L1 agents in hepatocellular carcinoma and renal cell carcinoma. Promising clinical activity has been demonstrated in patients with refractory GC/GEJC when treated with dual blockade combination with antiangiogenic agents and immune checkpoint inhibitors like PD-1/PD-L1 inhibitors in several phase I/II trials. This review highlights the trials investigating these novel combinations as well as their preclinical rationale.

## Background

Despite immune checkpoint inhibitors (IO) demonstrating durable responses and prolonged survival across multiple tumor types, the proportion of metastatic cancer patients who benefit from IO remains limited including in gastric and gastroesophageal cancer (GC/GEJC). Of note, given the similar underlying pathogenesis and genomic alterations between GC and GEJC, they have been considered and managed as one clinical entity and thus will be discussed here as one cancer type [1]. In locally advanced and metastatic GC/GEJC, although monotherapy IO has demonstrated benefit in the subset of patients with mismatch-repair/microsatellite instability high (MMR/MSI-H) as well as in in the 3rd line setting in patients with high programmed death ligand-1 (PD-L1) expression, benefit is largely limited in the rest of the population [2, 3]. A great effort has been focused on developing novel strategies to extend clinical benefit to non-responder populations, and one of the strategies is the combination of IO with other systemic therapeutic modalities such as molecularly targeted agents.

Primary resistance to IO therapy is explained in part by the immune suppressive nature of the tumor microenvironment (TME). The immune suppressive TME is characterized by increased infiltration of immunosuppressive cells including regulatory T cells (Treg), myeloid-derived suppressor cells (MDSCs), tumor-associated macrophages (TAMs), particularly of the tumor-promoting M2 phenotype, all of which consequently decrease the activity of immune effector cells such as cytotoxic T cells and helper T cells via multiple mechanisms such as production of immune suppressive cytokines and chemokines as well as derangement of metabolic pathways [4]. While the biological mechanisms leading to the establishment of the immune suppressive TME is multifactorial and complex, one of the best understood and most important mechanisms among them has been the effects of neo-angiogenesis [5].

The best-established biological mediator of tumor angiogenesis with concomitant immune suppressive effects is vascular endothelial growth factor (VEGF), a cytokine induced by local tissue hypoxia and acidosis, which promotes the growth of defective and leaky tumor vasculature [6]. In addition to the indirect effects on anti-tumor immunity by hindering the tumor infiltration of immune effector cells via its effects on the vasculature, VEGF has direct local and systemic immune suppressive effects [7, 8]. For example, VEGF is associated with increased infiltration of Treg, MDSCs, and M2 type TAMs into the tumor stroma and anti-VEGF therapy leads to reversal of these immune suppressive mechanisms [9–12]. Therefore, anti-VEGF therapy is associated with ‘normalization’ of the TME and has the potential to reverse therapeutic resistance to IO and is a promising therapeutic partner in a combination regimen.

Combination VEGF and PD-1/PD-L1 axis blockade has shown benefit in various tumors and is emerging as a promising combination strategy. A prominent and successful example is the IMBrave150 trial, in which bevacizumab (anti-VEGF-A) with atezolizumab (anti-PD-L1) demonstrated superior overall survival (OS) over sorafenib, the standard of care first-line systemic therapy for metastatic hepatocellular carcinoma (HCC) [13]. Furthermore, preliminary results of phase I/II trials of the multi-cohort COSMIC-312 trial has demonstrated promising potential of cabozantinib, a multi-receptor tyrosine kinase (TKI) inhibitor with VEGF inhibitory activity in combination with atezolizumab in metastatic HCC, non-small cell lung cancer, renal cell cancer (RCC), and prostate cancer [14–17]. Herein, we summarize the pre-clinical evidence supporting the synergy of anti-VEGF and anti-PD-1/PD-L1 in metastatic GC/GEJC and review the results of ongoing clinical trials evaluating this regimen to demonstrate that this emerging combination treatment has promising potential to advance the treatment paradigm of metastatic GC/GEJC.

## Search methodology

A systematic review was conducted according to PRISMA guidelines with the last search update being on December 17, 2020. The search was conducted in PubMed as well as major conference proceedings (ASCO, ESMO) using the following search query: (gastric cancer OR esophageal cancer OR gastroesophageal cancer OR gastroesophageal junction cancer OR esophageal adenocarcinoma) AND (VEGF OR anti-VEGF OR VEGF TKI) OR (IO OR ICI OR immune therapy OR anti-PD-1 OR anti-PD-L1). Additionally, clinicaltrials.gov was searched to identify ongoing trials that have not yet published reports. Studies were included if it evaluated a combination checkpoint inhibitor and VEGF targeted agent in a clinical trial setting in advanced GC/GEJC patients and reported efficacy or toxicity outcomes. Studies were excluded if it evaluated monotherapy checkpoint inhibitor or VEGF targeted agent, if it was a protocol-only publication without data, or if it reported overlapping data. In the latter case, the study with the most recent and/or most comprehensive data was included.

The initial search identified a total of 1125 studies. After review by title and abstract, 45 studies remained. Additionally, 3 studies were added via reference review. Following full-text review, 7 studies were included in the final review. 12 additional studies were identified via clinicaltrials.gov.

### Antiangiogenic therapy in gastroesophageal cancers

Bevacizumab was tested in the first line setting in treatment of metastatic GC patients in combination with chemotherapy compared with chemotherapy alone. The combination showed advances in response rates (46% vs 37%) and PFS (6.7 vs 5.3 months). Unfortunately, the benefit of OS was not statistically significant (12.1 vs 10.1 months) [18].

On the other hand, the survival benefit was demonstrated with ramucirumab, the anti-VEGFR2 antibody, either as monotherapy versus best supportive care (5.2 vs 3.8 months) or in combination with paclitaxel versus paclitaxel alone (9.6 vs 7.4 months) in the second-line setting, which validated the role of VEGF/VEGFR-2 signaling as an important therapeutic target in advanced GC/GEJC and esophageal adenocarcinomas [19, 20].

In addition, VEGFR TKIs have also been explored in patients with GC/GEJC. Regorafenib, was tested in the phase II randomized placebo-controlled trial (INTEGRATE). The trial enrolled 147 evaluable patients. A significant improvement was seen in median PFS, 2.6 versus 0.9 months favoring regorafenib with a hazard ratio of 0.4 [21].

Cabozantinib, another multi-TKI inhibitor targeting VEGFR2, MET, RET, and AXL showed broad single agent activity in multiple solid tumors including gastric cancer, and is currently FDA approved for patients with RCC and HCC after progression on sorafenib. A Phase 2 randomized discontinuation trial evaluated the efficacy and safety of cabozantinib in 526 patients with 9 tumor types, including a cohort of 21 patients with GC. Patients received cabozantinib 100 mg orally once daily over a 12-week lead-in phase. ORR, the primary endpoint of the lead-in phase at week 12 for the GC cohort was 10% and the disease control rate was 33%. The most common grade 3 or higher adverse events included fatigue, diarrhea, hypertension, and palmar-plantar erythrodysesthesia [22]. (Table 1).

**Table 1:** Selected trials for monotherapy checkpoint inhibitor and VEGF TKI in unresectable gastroesophageal cancer

Trial	Interventions	Phases	Treatment setting	Results
NCT00548548	Bevacizumab + CTx versus CTx	III	1st line	ORR: 46 versus 37% PFS: 6.7 versus 5.3 mo OS: 12.1 versus 10.1 mo Most common grade 3–4 AE: neutropenia (35%), anemia (10%), anorexia (8%)
REGARD RAINBOW	Ramucirumab versus BSC Ramucirumab + CTx versus CTx	III	2nd line	OS: Ramucirumab versus BSC: 5.2 versus 3.8 mo Ramucirumab + CTx versus CTx: 9.6 versus 7.4 mo Grade 3–4 AEs: 57% versus 58% (REGARD) Grade 3–4 AEs: 47% versus 42% (RAINBOW)
INTEGRATE	Regorafenib versus placebo	II	2 or fewer lines of Tx	PFS: 2.6 versus 0.9 mo Grade 3–4 AE: 67% versus 52%
NCT00940225	Cabozantinib	II	Advanced, recurrent, or metastatic	ORR: 10% DCR: 33% Grade 3–4 AE: 74.5%
KEYNOTE-59	Pembrolizumab	II	3rd line PD-L1 +	ORR: 15.5% mDOR: 16.3 mo Grade 3–5 AE: 17.8%
KEYNOTE-62	Pembrolizumab or Pembrolizumab + CTx versus CTx	III	1st line	mOS: 10.6 versus 11.1 mo (CPS 1 or higher) PFS: 6.9 versus 6.4 mo (CPS 1 or higher) Grade 3–4 AE: 17% versus 73% versus 69% (Pembro versus Pembro + CTx versus CTx)
KEYNOTE-180	Pembrolizumab	II	3rd line	Overall ORR: 9.9% mDOR: NR (1.9–14.4 mo) Grade 3–4 AE: 12.4%
KEYNOTE-61	Pembrolizumab versus Paclitaxel	III	2nd line	mOS: 9.1 versus 8.3 mo mPFS: 1.5 versus 4.1 mo Grade 3–5 AE: 14% versus 35%
KEYNOTE-181	Pembrolizumab versus CTx	III	2nd line	mOS: 9.3 versus 6.7 mo (CPS 10 or higher) Grade 3–5 AE: 18.2% versus 40.9%
KEYNOTE-590	Pembrolizumab + CTx versus CTx	III	1st line	mOS: 12.4 versus 9.8 mo mDOR: 8.3 versus 6.0 mo Discontinuation rates due to AEs: 19% versus 12%
CheckMate-649	Nivolumab + CTx versus CTx	III	1st line	mOS: 13.8 versus 11.6 mo (overall) mOS: 14.0 versus 11.3 mo (CPS 1 or higher) mOS: 14.4 versus 11.1 mo (CPS 5 or higher) Grade 3–4 AEs: 59% versus 44%
CheckMate-32	Nivolumab w/wo ipilimumab	I/II	2nd line	ORR 12%, 24% 12-mo OS: 39%, 35% Grade 3–4 AEs: 17% versus 47% versus 27%*
ATTRACTION-2	Nivolumab versus placebo	III	2nd line	mOS: 5.3 versus 4.1 mo 12-mo OS: 26.2 versus 10.9% Grade 3–4 AEs: 10% versus 4%
JAVELIN Gastric 300	Avelumab versus CTx	III	3rd line	mOS: 4.6 versus 5.0 mo PFS: 1.4 versus 2.7 mo ORR: 2.2 versus 4.3% Grade 3–4 AEs: 9.2% versus 31.6%
NCT02340975	Durvalumab w/wo ipilimumab	I/II	2nd line	ORR: 0%, 7.4% 12 mo OS: 4.6%, 37% Grade 3–4 AEs: 17% versus 4%, versus 42% versus 16%**

^*^Nivolumab only versus Nivolumab 1 mg/kg + ipilimumab 3 mg/kg versus nivolumab 3 mg/kg + ipilimumab 1 mg/kg

^**^Durvalumab + tremelimumab in 2nd line setting versus durvalumab only versus trememlimumab only versus durvalumab + tremelimumab in 3rd line setting

### Immune checkpoint inhibitor agents in gastroesophageal cancers

#### Anti-PD-1 ± anti-CTLA-4 agents

 The phase IB KEYNOTE-028 trial was the first study to demonstrate the safety and treatment response of the anti-PD-1 antibody pembrolizumab in unresectable esophageal cancer patients with PD-L1 expression who received two or more lines of prior treatment (ORR 30, median duration of response (mDOR) 15 months) [23]. This study was followed by the phase II KEYNOTE-059 trial which confirmed the efficacy of pembrolizumab in the PD-L1 + population (ORR 15.5%, mDOR 16.3 months) and led to the FDA approval of pembrolizumab in PD-L1 + advanced GC/GEJC patients who progressed on two or more lines of therapy. Of note, favorable results were seen only in the PD-L1 + and not in the PD-L1-population with PD-L1 expression assessed by the combined positive score (CPS) [3].

The phase I/II CheckMate-032 trial demonstrated the potential efficacy of nivolumab alone or in combination with ipilimumab (ORR 12%, 24%; 12-month OS 39%, 35% in N and N + I respectively) in advanced GC/GEJC patients who progressed on at least one line of previous therapy [24]. Additionally, in the ATTRACTION-2 trial, nivolumab demonstrated superior median OS (5.3 vs. 4.1 months, P < 0.0001) and 12-month OS rate (26.2 vs. 10.9%) in biomarker non-selected patients with relapsed or refractory unresectable GC/GEJC in an Asian population leading to the nivolumab’s approval in Japan, Taiwan, and South Korea [25].

On the other hand, trials of anti-PD-1 agents have also shown comparably disappointing results, suggesting novel strategies are necessary to overcome primary or secondary resistance to therapy. For instance, the phase III KEYNOTE-062 trial failed to demonstrate the superiority of pembrolizumab compared to chemotherapy in the frontline setting with pembrolizumab failing to show superiority to chemotherapy even in the PD-L1 > 10% CPS group [26]. Furthermore, the phase II KEYNOTE-180 trial demonstrated a disappointing tumor response of pembrolizumab in the third line setting (ORR 5.2%) in GEJ cancer [27]. Likewise, the phase III KEYNOTE-181 trial failed to show improved OS of pembrolizumab over chemotherapy in the intention-to-treat population of patients with unresectable or advanced stage esophageal cancer who progressed on first line therapy [28]. Lastly, the phase III KEYNOTE-061 trial failed to show OS benefit of pembrolizumab over chemotherapy in the second line setting in non-selected GC/GEJC population [29].

Despite the underwhelming results of anti-PD-1 therapy alone in the frontline settings, anti-PD-1 therapy in combination with chemotherapy has demonstrated favorable results in selected populations. Namely, the phase III KEYNOTE-590 trial demonstrated the superior median OS (median OS 12.4 vs 9.8 months; HR, 0.73, 95% CI, 0.62–0.86) and PFS (median 6.3 vs 5.8 months; HR 0.65; 95% CI, 0.55–0.76) of pembrolizumab with chemotherapy versus chemotherapy alone in the overall population which was comprised of 73% esophageal squamous cell carcinoma and 27% esophageal adenocarcinoma. Of note, the OS benefit (median 13.5 vs 9.4 months; HR 0.62; 95% CI, 0.49–0.78) and PFS (median 7.5 vs 5.5 months; HR 0.51; 95% CI, 0.41–0.65) was greater in the CPS ≥ 10 subpopulation [30]. Similarly, nivolumab with chemotherapy demonstrated superior efficacy compared to chemotherapy alone in the phase III CheckMate-649 trial. Of note, the patient population comprised solely of GC/GEJC patients and enriched with the subgroup of patients with PD-L1 CPS > 5% constituting > 50% of the total study population. Nivolumab plus chemotherapy demonstrated superior median OS in the overall population (HR 0.80, 99.3% CI, 0.68–0.94). Of note and as expected, toxicity was higher in the nivolumab plus chemotherapy group (grade 3–4 treatment-related adverse events, 59 vs. 44%) [31]. Taken together, these studies suggest that in selected populations, appropriate combination regimens with IO therapy may overcome therapeutic resistance. (Table 1).

#### Anti-PD-L1 ± anti-CTLA-4 agents

In a phase I/II trial (NCT02340975) of advanced stage GC/GEJC patients who progressed on first-line therapy, durvalumab alone or in combination with tremelimumab showed modest anti-tumor and survival outcomes (ORR 0%, 7.4%; 12-month OS, 4.6%, 37% for durvalumab and durva plus treme respectively) [32]. Another PD-L1 inhibitor, avelumab, was explored in the phase III JAVELIN Gastric 300 trial comparing avelumab vs physician’s choice of chemotherapy. This trial did not meet its endpoint for improvement in OS or PFS compared to chemotherapy in the third line setting for advanced GC/GEJC [33]. Subgroup analysis based on certain molecular markers like PD-L1 was not done as in several trials that explored PD-1 inhibitors. Collectively, the overall outcome of monotherapy with PD-L1 and PD-1 inhibitors suggest that combinatorial approaches with appropriate partners would potentially lead to better efficacy and survival in this patient population. (Table 1).

### Angiogenesis and the tumor immune microenvironment

There is extensive evidence that angiogenesis pathways are able to foster an immune suppressive tumor microenvironment (TME) in various ways including through direct suppression of antigen presenting cells and immune effector cells or through augmentation of immune suppressive cells such as Treg, MDSCs, and TAMs.

VEGF-driven angiogenesis involving its cognate receptors VEGFR1-3 is among the most important angiogenic factors associated with multi-pronged immune suppressive effects in the TME [34–36]. For example, binding of VEGF to its receptors on DCs inhibits their maturation and antigen presentation and induces PD-L1 expression on the cell surface [37]. Furthermore, increased VEGF levels leads to inhibition of cytotoxic T cell trafficking, proliferation, and effector function [9, 38, 39]. In addition, VEGF drives the expansion of Tregs and MDSCs as well as TAMs in the TME [40]. Moreover, angiogenic pathways other than VEGF have also been shown to contribute to immune suppression in the TME. For example, Ang-2 is a cytokine ligand of the receptor Ang1/Tie2, which modulates VEGF-mediated angiogenesis [41, 42]. Ang-2 expression leads to increased endothelial adhesion of TIE-2 expressing monocytes/macrophages and stimulates their production of immunosuppressive cytokines such as IL-10 [43, 44].

In turn, targeting VEGFR using various approaches including VEGF multi-TKIs have been found to promote an immune permissive TME by normalizing vascularization and reducing MDSCs (CD11b + , Gr +). In addition, VEGF TKIs have been described to have immuno-modulatory effects in vitro and in murine models for several cancers. For instance, cabozantinib appears to drive its impact on Tregs via the HGF/c-Met pathway, where this receptor signaling cascade moderates several immune cell functions [45]. HGF was shown to induce Tregs (CD4 + CD25 + FoxP3) via hindering of DC function in a murine central nervous system autoimmunity model [46]. HGF cultured monocytes differentiate into monocytic cells that generate soluble factors (e.g., IL-10) which are known to esteem immune suppressive conditions ideal for tumor development [47].

The immunomodulatory effects of cabozantinib have been studied mostly in in vitro and in vivo tumor models other than GC/GEJC. For example, in murine colorectal cancer tumor cells, cabozantinib led to an increase in major histocompatibility complex (MHC) class 1 and cell surface molecules FAS expression, as well as promoting intercellular adhesion molecule 1, and calreticulin expression, which together heighten immune cell recognition and enhance sensitivity to T-cell mediated lysis [48]. Furthermore, treatment with cabozantinib boosted peripheral CD8 + T cells and reduced Treg and MDSCs in the spleen. In murine tumor models, marked tumor infiltration of CD8 + T cells and Tregs along with a reduction in tumor infiltration of MDSCs and TAMs was seen. These results highlight that cabozantinib alleviates the immune suppressive environment in mouse tumors. In a chimeric murine model of metastatic castrate resistance prostate cancer, a modest effect on tumor mass was seen when cabozantinib was used as single agent, but in combination with anti-CTLA-4 or anti-PD-1 a robust synergistic response was noted, mediated by neutralization of MDSCs (CD11b + , Gr1 +) [49]. PI3K signaling was also found to be hampered by cabozantinib, which impaired the release of cytokines by prostate cancer cells; which in turn prompted expression of MDSC genes responsible for tumor suppressive activity. The negative modulatory impact on GR1 + MDSCs was associated with an increase in CD8 + T cell tumor infiltration in prostate tumors, which endorses the antagonistic activity of Gr1 + MDSCs on the CD8 + population of T cells.

**Fig. 1:**
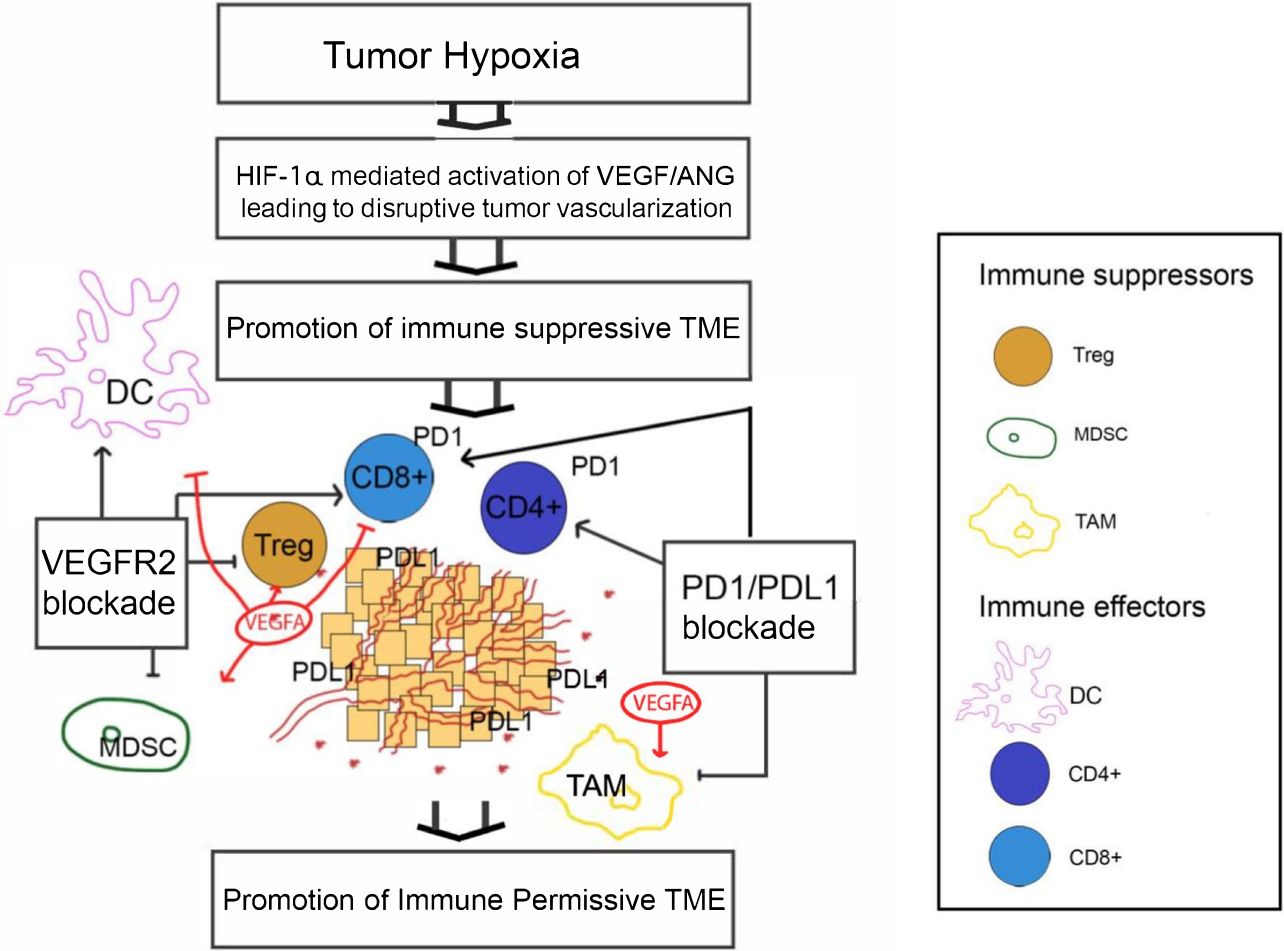
Rationale for combining VEGFR2 and PD-1/PD-L1 blockade. Tumor hypoxia leads to VEGFA/ANG induced disruptive tumor vascularization in a HIF-1a dependent manner. In turn, an immunosuppressive TME becomes established characterized by Treg, MDSCs, and M2 TAMs which inhibit CD4+ and CD8+ effector T cell mediated tumor immune response. In addition, the PD-1/PD-L1 axis is central in maintaining the immune suppressive TME by inhibiting CD4+ and CD8+ effector T cells. VEGFR2 blockade leads to tumor vessel normalization which potentiates PD1/PDL1 blockade by reversing established immune suppressive mechanisms. VEGFR2, vascular endothelial growth factor receptor 2; PD-1/L-1, programmed-death-1/ligand-1; ANG, angiopoietin; HIF-1a, hypoxia inducible factor-1a; Treg, regulatory T cell; MDSC, myeloid derived suppressor cell; TAM, tumor associated macrophages; DC, dendritic cells

In addition to cabozantinib, other VEGF TKIs have shown immunomodulatory effects in both in vitro and in vivo studies, further supporting the potential role of VEGF TKIs in combination IO therapy. For instance, lenvatinib has been associated with reduction of TAMs in multiple tumor models and increased tumor infiltration of interferon-γ producing CD8 + T cells as well as memory T cells [50–52]. Furthermore, regorafenib has also been associated with decreased infiltration of TAMs and increased M1 macrophages in colorectal tumor models [53] (Fig. 1).

### Combined targeting of VEGFR2 and immune checkpoint inhibition in gastroesophageal cancers

#### Anti-PD-1/PD-L1 combinations with VEGFR2 monoclonal antibody

Recent data showed encouraging activity with ramucirumab plus nivolumab, ramucirumab plus pembrolizumab, and ramucirumab plus durvalumab in GC/GEJC patients.

The results of the phase I/II NivoRam study were recently presented [54]. In this trial, ramucirumab plus nivolumab was evaluated in the 2nd line setting for advanced gastric cancer patients. Primary endpoints were dose limiting toxicities (DLT) and 6-month PFS. Patients underwent evaluation of PD-L1 using the CPS score and 44% were determined to be PD-L1 + with a cutoff of 1 or higher. Six patients were evaluated in the phase I part while an additional 40 patients were subsequently added in the phase II part. ORR was 26.7% and disease control rate (DCR) was 62.2%. No dose limiting toxicities were observed in the phase I part. The 6-month PFS was 37.4% (90% confidential intervals: 25.7–49.2%), which met the primary endpoint of the phase II section.

The similar combination of ramucirumab plus pembrolizumab was recently explored in a multi-cohort phase Ib trial in 3 tumor types including previously treated GC/GEJC (n = 41). The primary endpoints were DLTs and adverse event incidence. Tumors were evaluated for PD-L1 TPS, MMR/MSI, and HER2 statuses. The combination showed manageable safety profile with favorable ORR of 7% and DCR of 44% [55]. Furthermore, ramucirumab in combination with durvalumab was evaluated in a single-arm phase Ia trial which included three cancer types including GC/GEJC (n = 29). This regimen demonstrated a promising tumor response (ORR 21%) and survival (OS 2.6 months; PFS 12.4 months) [56]. (Table 2).

**Table 2:** Ongoing clinical trials evaluating checkpoint inhibitor plus VEGF TKI in unresectable gastroesophageal cancer

NCT number	Status	Interventions	Phases	Treatment setting	Results
NCT02999295	Completed	Nivolumab + ramucirumab	Phase 1/2	2nd line	ORR: 26.7% DCR: 62.2% Grade 3–4 AE: 33/46 No deaths
NCT03406871	Active, not recruiting	Nivolumab + regorafenib	Phase 1	3rd line	ORR: 44% mPFS: 5.6 mo mOS: 12.3 mo Grade 3–4 AEs: 20/50 3 DLTs with Rego 160 mg
NCT02572687	Active, not recruiting	Durvalumab + ramucirumab	Phase 1	2nd line	ORR: 21% mOS: 2.6 mo mPFS: 12.4 mo Grade 3–4 AE: 37.9%
NCT02443324	Active, not recruiting	Pembrolizumab + ramucirumab	Phase 1	4th line	ORR: 7% DCR: 44% Grade 3–4 AE: 11/29
NCT03609359	Active, not recruiting	Pembrolizumab + lenvatinib	Phase 2	1st of 2nd line	ORR: 69% DCR: 100% mPFS: 6.9 mo Grade 3–4 AE: 14/29 No Gr 4–5 AE
NCT02942329	Unknown	SHR1210 + apatinib	Phase ½	2nd line	ORR: 16% DCR: 78% Grade 3–4 AE: 20/33* 4/43 DLTs
NCT03539822	Recruiting	Cabozantinib + durvalumab	Phase 1/2	3rd line	ORR: 25% DCR: 85% Median time to progression: 16 weeks No DLTs
NCT04267549	Recruiting	Sintilimab + apatinib + chemotherapy	Phase 2	2nd line	Not yet reported
NCT04182724	Recruiting	Camrelizumab + apatinib + nab-paclitaxel	Phase 1/2	2nd line	Not yet reported
NCT04006821	Recruiting	Camrelizumab + apatinib	Phase 2	2nd line	Not yet reported
NCT03995017	Recruiting	Rucaparib + ramucirumab + nivolumab	Phase 1/2	2nd line	Not yet reported
NCT03966118	Recruiting	Avelumab + ramucirumab + paclitaxel	Phase 2	2nd line	Not yet reported
NCT03813784	Recruiting	SHR-1210 + capecitabine + oxaliplatin then shr-1210 + apatinib versus capecitabine + oxaliplatin	Phase 3	1st line	Not yet reported
NCT03797326	Recruiting	Pembrolizumab + lenvatinib	Phase 2	3rd line	Not yet reported
NCT03603756	Recruiting	SHR-1210 + apatinib + chemotherapy	Phase 2	1st line	Not yet reported
NCT03475953	Recruiting	Regorafenib + avelumab	Phase 1/2	2nd line	Not yet reported
NCT03321630	Recruiting	Lenvatinib + pembrolizumab	Phase 2	2nd line	Not yet reported
NCT03170960	Recruiting	Cabozantinib + atezolizumab	Phase 1/2	2nd line	Not yet reported
NCT02734004	Active, not recruiting	MEDI4736 + olaparib ± bevacizumab	Phase 1/2	2nd line	Not yet reported

^*^Toxicity results of overall population including patients with hepatocellular cancer patients. Grade 3–4 AEs represent incidence of patients receiving recommended phase II trial dose

#### Anti-PD-1/PD-L1 combinations with VEGFR multi-TKIs

The combination of VEGFR TKI plus checkpoint inhibitors has been explored in single arm studies and showed early signals of promising efficacy, in various tumor types including GC/GEJC.

A relevant example is the VEGFR multi-TKI regorafenib plus nivolumab in chemotherapy-refractory gastric cancer which yielded ORR of 44% compared to ORR of 3% previously seen with regorafenib alone in the INTEGRATE trial [57]. Furthermore, median PFS and OS were 5.6 and 12.3 months respectively. The incidence of grade 3 or higher treatment-related adverse events was 40% in the overall population with rash, proteinuria, and palmoplantar erythrodysesthesia the most common among them.

Lenvatinib (VEGFR multi-TKI) was also recently combined with pembrolizumab in chemo-refractory and chemo-naïve gastric and gastroesophageal adenocarcinoma patients (14 pts were chemo naïve and 15 pts were chemo refractory) and yielded a primary endpoint of ORR of 69% and DCR of 100%. Median PFS was 6.9 months in the overall study population [18, 58]. In this study, tumor specimens were evaluated for MMR/MSI, HER2, EBV, PD-L1 CPS, and TMB statuses. The combination regimen demonstrated a favorable toxicity profile with no grade 4 or 5 treatment-related adverse events and grade 3 events occurring in 48% of patients. The most common grade 3 treatment-related adverse events were hypertension, proteinuria, and thrombocytopenia.

Apatinib (VEGFR2 inhibitor) in combination with SHR-1210 (anti-PD-1 antibody) has also been evaluated in a phase I/I, multi-cohort trial with advanced gastric and hepatocellular cancer patients. The primary endpoint of this trial was overall survival at 6 and 12 months. Interim results showed moderate overall toxicity with incidence of DLTs in the overall population of 26.7% (4/43) and grade 3–4 adverse events of 60% (20/33) in patients who received the recommended phase II dose. The confirmed objective response was 16% (4/25) in the GC/GEJC population and DCR 78% [59].

Saeed et al. are currently testing cabozantinib plus durvalumab in the phase I/II CAMILLA trial primarily targeting advanced pretreated gastric and esophageal adenocarcinoma patients. Primary outcomes of this study are maximum tolerated dose which is defined as the highest dose studied for which the observed incidence of DLT is less than 33% and ORR. The study is ongoing, but phase I results are encouraging. There were no dose-limiting toxicities when the cabozantinib dose was increased from 20 to 60 mg. Partial responses (PR) seen in 5 of 20 (25%) patients and DCR (partial response + stable disease) seen in 17 of 20 (85%) patients treated with the combination. Median time to disease progression (PD) was 16 weeks (range 8–40 +) [60] (Table 2).

### Challenges and future directions

Although both VEGFR inhibitors and PD-1/PD-L1 inhibitors have demonstrated some activity as single agents in the treatment of GC and GEJC, the mechanisms by which the VEGFR plus PD-1/PD-L1 targeting combinations lead to benefit and predictors of response to therapy need further investigations. To date, PD-L1 expression, high TMB and MSI-High status correlated with treatment response and improved survival from monotherapy with PD-1/PD-L1 blockade. Both PD-L1 expression using the CPS score and MSI status have also been validated as predictors of response to PD-1 plus chemotherapy combinations in this population as per the CheckMate-649 trial, KEYNOTE-590, and others. Ongoing and future trials testing the VEGFR plus PD-1/PD-L1 inhibitors should test the predictive value of those similar markers as well as explore additional predictive & prognostic markers which will add essential data to the field supporting personalized medicine in immuno-oncology. There remains a challenge in the interpretation of biomarkers and evaluation of their predictive values in the setting of combination immune and angiogenesis blockade. Future studies focusing on biomarker development aimed at predicting synergy between IO and VEGF targeting agents are warranted. Considering the toxicity profile of this novel combination, which is less favorable then either drug alone, tailoring this strategy to the population that would benefit most would be ideal.

Furthermore, results from early phase trials testing VEGFR plus PD-1/PD-L1 inhibitors in advanced stage GC/GEJC suggest that using this therapeutic approach in earlier vs later lines setting is associated with more favorable tumor responses and survival outcomes. This could theoretically be related to more immune-permissive tumor microenvironment in early line settings and encourages future direction to validate those results in larger pivotal phase III trials. Such trials could test this combinatorial strategy alone in sequential approach post chemotherapy or in combination with chemotherapy. If the survival advantage of such combination is confirmed in phase III trials, a next step would be the incorporation of this therapy with multimodality approaches in early stage GC/ GEJC.

## Conclusion

Antiangiogenic therapy in GC/GEJC patients appears to be a promising option to overcome resistance to IO therapy by modulating the anti-tumor immune response. The preclinical and clinical evidence reported to date suggest that combination IO with a VEGF targeting agent may improve the overall outcome. Mature survival results from ongoing trials are highly anticipated and much awaited.

## Data Availability

Not applicable.

## Abbreviations

IO: Immune checkpoint inhibitor
GC/GEJC: Gastric cancer/gastroesophageal cancer
MMR/MSI-H: Mismatch repair/microsatellite instability high
PD-1/PD-L1: Programmed death/programmed death ligand-1
TME: Tumor microenvironment
Treg: Regulatory T cell
MDSC: Myeloid derived suppressor cell
VEGF: Vascular endothelial growth factor
OS: Overall survival
HCC: Hepatocellular carcinoma
PFS: Progression free survival
VEGFR: VEGF receptor
TKI: Tyrosine kinase inhibitor
ORR: Objective response rate
mDOR: Median duration of response
CI: Confidence interval
CPS: Combined positive score
HR: Hazard ratio
CTLA-4: Cytotoxic T lymphocyte antigen-4
DC: Dendritic cell
CNS: Central nervous system
HGF: Human growth factor
MHC: Major histocompatibility complex
DCR: Disease control rate
